# Helmsmanship Leadership: Temporal Dynamics of High-Responsibility, Low-Visibility Governance

**DOI:** 10.3390/bs16050710

**Published:** 2026-05-05

**Authors:** Xiuxia Liu, Lunan Li, Hangyu Zhang, Qiuhua Lin

**Affiliations:** Department of Physical Education, Xiamen University, Xiamen 361005, China; 26920251153827@stu.xmu.edu.cn (L.L.); zhanghangyu@stu.xmu.edu.cn (H.Z.)

**Keywords:** leadership, helmsmanship, dragon-boat, high-responsibility, low-visibility

## Abstract

Leadership research has long focused on the “high-visibility, high-responsibility” heroic leadership paradigm, while systematically neglecting the critical quadrant of “low-visibility, high-responsibility” (HRLV) leadership. Through ethnographic observation and in-depth interviews with Chinese dragon-boat helmsters as an extreme case, this study constructs a Helmsmanship Leadership theoretical framework, revealing the core operating mechanisms of HRLV leadership. The framework comprises 3 interlocking dimensions: Fade—achieving silent coordination through sub-threshold interventions, decoupling influence from visibility; Fail-safe—safeguarding the system’s floor through preventive authority, with success marked by the “absence of disaster”; Fit—situational attunement and going with the flow, serving as a conduit for environmental forces rather than a controller. The systemic coupling of these 3 dimensions enables helmsters to sustain collective survival under extreme conditions of zero-error tolerance and high interdependence. This study decouples leadership from visibility, revealing how influence is generated under conditions of “not being seen.” It expands the theoretical boundaries of relational leadership, extending relationality from interpersonal interaction to the leader’s relationship with the system, with risk, and with the environment. It contributes to leadership theory a “visibility-responsibility” analytical framework, revealing the core characteristics and operating logic of the long-overlooked leadership form of “high-responsibility, low-visibility.” In doing so, it provides new analytical tools for understanding governance in high-risk, highly interdependent systems.

## 1. Introduction

Leadership styles have long occupied a central place in global management research. However, the theoretical discourse has been predominantly shaped by frameworks originating in North America and Europe. Prominent examples include charismatic leadership (House, 1976), transformational leadership (Bass, 1985), servant leadership (Greenleaf, 1977), and transactional or shared leadership models.

In these paradigms, leaders are typically cast as pivotal agents of organizational action. They occupy the spotlight, articulating compelling visions, energizing teams, and driving strategic change. Their success is measured by visible performance gains, and they receive public recognition accordingly. Conversely, when organizations face major failures or crises, these same leaders are often held primarily accountable. This “high-visibility, high-accountability” model reflects an emphasis on personal agency, control, and outcome orientation. It rests on an implicit assumption: the value of leadership lies chiefly in its capacity to raise the organization’s performance ceiling—continually pushing boundaries and achieving new peaks (Bass, 1985; Day, 2014; Pfeffer, 1977).

Yet a growing body of research challenges this assumption, arguing that leadership is deeply embedded in local systems of meaning (House et al., 2004; Dorfman et al., 2012). Currently, the “high-responsibility, low-visibility” leadership model struggles to explain leadership practices characterized by implicit authority, relational orientation, and collective harmony. For example, employees in individualistic cultures tend to favor charismatic leadership, whereas those in collectivistic settings—such as China—place greater value on leader humility and group cohesion (Kirkman et al., 2006). Similarly, Confucian norms emphasizing hierarchical order can render Western ideals of empowerment or participative decision-making culturally incongruent (Farh & Cheng, 2000).

These limitations reveal a critical blind spot: dominant theories overemphasize leaders as visible drivers of performance “ceilings” while overlooking leadership forms situated in the low-visibility, high-accountability quadrant—where leaders remain embedded within the collective, claim no credit, seek no recognition, yet bear full responsibility when the system teeters on collapse.

More broadly, this blind spot also reflects a knowledge–production imbalance in management research. Indigenous management scholars have long argued that theories derived primarily from Euro-American settings cannot simply be treated as universally exhaustive, and that high-quality theorizing from Chinese contexts should not be limited to contextual “applications” of Western models, but should generate original concepts from local cultural and organizational realities (Tsui, 2004; Li et al., 2012). In the leadership domain specifically, traditional Chinese philosophies provide not merely a cultural backdrop but potential theoretical micro-foundations for understanding authority, relationality, and action in ways insufficiently captured by mainstream Western constructs (Ma & Tsui, 2015).

This gap is especially pronounced across generations. Empirical studies with university students across East and Southeast Asia consistently show that, regardless of nationality, young respondents identify listening, collaboration, ethical exemplarity, and service to others as core attributes of ideal leadership. They widely regard assertive, directive styles as outdated (Wright et al., 2021). This reflects a deeply rooted expectation of moral authority in the Sinic cultural sphere—one centered on virtue and relational integrity rather than individual heroism or transformative vision.

Notably, even amid globalization and digital transformation, Asian youth continue to anchor their conceptions of leadership in indigenous ethical frameworks (Nor et al., 2017).

To address this gap, scholars have called for recentering “place” in leadership research—emphasizing that leadership emerges within specific constellations of relational networks, symbolic systems, and spatial contexts (Collinge & Gibney, 2010; Collinge et al., 2010; Mabey & Freeman, 2010). As Collinge and Gibney (2010, p. 388) argue, place constitutes the “relational soil in which interpretive leadership activities unfold across levels and boundaries.”

Yet empirically grounded, embodied exemplars of such situated leadership remain scarce—particularly those that move beyond abstract constructs like paternalistic or humble leadership to reveal observable, context-specific practices. Several adjacent constructs partially illuminate this terrain, but none fully capture the phenomenon at stake here. Quiet and invisible leadership foregrounds low-profile influence and the practical invisibility of consequential leadership work (Badaracco, 2002; Alvesson & Sveningsson, 2003). Silent coordination points to collective alignment achieved with minimal explicit communication (Rico et al., 2008). HRO and sensemaking, in turn, highlight reliability, error prevention, and the preservation of order under uncertainty (Weick, 1993; Weick et al., 1999; Maitlis & Christianson, 2014). Taken together, however, these perspectives stop short of theorizing a leadership position in which invisibility of credit is systematically paired with concentration of responsibility, and in which effective action depends on deliberate self-effacement rather than visible direction. This raises a critical theoretical question: How can we conceptualize a form of leadership that operates without visibility or personal acclaim, yet reliably safeguards the system’s integrity in moments of crisis?

Chinese dragon-boat helmsters provide an extreme case in which the asymmetry between concentrated responsibility and low visibility is especially pronounced. In this setting, helmsters may bear disproportionate blame for failure while receiving limited public recognition for success, all while making continuous micro-adjustments to rhythm, direction, and safety from the rear of the boat. This makes the setting analytically valuable for examining a form of leadership that is consequential yet not organized around public prominence.

Accordingly, this study does not seek to overturn existing leadership theories or to claim a universally applicable new paradigm. Rather, it uses an extreme Chinese case to develop an interpretive framework for understanding how visibility, credit, and responsibility may come apart under particular cultural and relational conditions. By doing so, the paper aims to contribute a culturally grounded concept that can inform broader theoretical discussion while remaining appropriately bounded by the specificity of the empirical setting.

## 2. Methods

### 2.1. Research Paradigm and Design

This study adopts a constructivist paradigm, which posits that reality is socially constructed and that knowledge emerges through the interactive engagement between researcher and participants (Crotty, 1998). Guided by this epistemological stance, we employ an interpretive qualitative research design to explore in depth how dragon-boat helmsters enact leadership within their specific cultural context and to understand the underlying logics that shape their practices. This approach enables us to move beyond observable behaviors and uncover the lived meanings participants ascribe to their roles, responsibilities, and relational dynamics.

More specifically, this study can be understood as an extreme-case qualitative inquiry. Chinese dragon-boat helmsters represent a theoretically revealing manifestation of the phenomenon of interest, because the asymmetry between concentrated responsibility and low visibility is amplified in this setting. Prior methodological scholarship suggests that extreme or revelatory cases are particularly useful for inductive theory building, as they make underlying social mechanisms more visible under rare or intensified conditions (Flyvbjerg, 2004; Eisenhardt & Graebner, 2007). Accordingly, this setting was selected strategically to illuminate the core logic of high-responsibility, low-visibility leadership in a more analytically tractable way.

### 2.2. Participants and Sampling

This study employed purposive sampling to ensure participants possessed substantial experience as dragon-boat helmsters and could provide rich, in-depth insights into the role (Palinkas et al., 2015). Participants were current or former helmsters from dragon-boat teams across different regions of China.

Sampling criteria included: (a) at least one year of formal helmster experience, and (b) participation in provincial-level or higher dragon-boat competitions. To enhance data richness and capture diverse perspectives, we sought heterogeneity in age, gender (where applicable), and team background.

A total of 12 helmsters were ultimately recruited. Their demographic and experiential profiles are summarized in Table 1. All participants provided written informed consent after receiving a full explanation of the study’s purpose and procedures.

### 2.3. Data Collection

Data were primarily collected through semi-structured in-depth interviews. The interview guide was organized around core themes, including participants’ role conceptions, sources of authority, decision-making processes, interactions with crew members, psychological responses to pressure and errors, and their understandings of leadership within the Chinese cultural context.

This flexible format ensured coverage of key research questions while allowing space for participants to share their unique experiences and perspectives (Kvale & Brinkman, 2009). All interviews were conducted in private, convenient locations chosen by participants to foster a safe and open dialogue. Each session lasted approximately 30 to 40 min and was audio-recorded using a digital voice recorder (Sony ICD-PX470, Sony Corporation, Tokyo, Japan) with the participant’s informed consent.

### 2.4. Data Analysis

Interview recordings were transcribed verbatim into text using speech-to-text transcription software (Xunfei Tingjian, iFLYTEK Co., Ltd., Hefei, China) and carefully checked against the audio files by the research team to ensure transcription accuracy. For data analysis, we employed constructivist thematic analysis (Braun & Clarke, 2019), which is well-suited to exploring patterned meaning across participants’ accounts while recognizing that themes are interpretive productions rather than objective entities simply waiting to be discovered. The analysis was facilitated using qualitative data analysis software (NVivo 12 Plus, QSR International, Melbourne, Australia).

The analysis proceeded iteratively rather than linearly. First, all transcripts were read repeatedly by the first author to achieve deep familiarization with the dataset, while preliminary analytic notes were recorded regarding recurring expressions, tensions, and role-related meanings. Second, meaningful segments of data were assigned initial codes that stayed close to participants’ language and experience. Third, related codes were clustered into broader candidate themes that captured recurrent patterns in how helmsters understood responsibility, invisibility, risk, and situational adaptation. Fourth, these candidate themes were reviewed against both the coded extracts and the full dataset to assess their internal coherence and external distinctiveness. Some codes were merged, refined, or discarded during this stage. Fifth, the themes were further defined and named to clarify their conceptual scope and analytical contribution. Finally, representative quotations were selected to illustrate each theme and to make explicit the link between empirical material and interpretive claims.

Interviewing and analysis were conducted in parallel. As data collection progressed, newly generated interviews were compared with earlier transcripts to examine whether substantially new conceptual insights were still emerging. By the final interviews, no new patterns that significantly altered the developing thematic structure were identified, and the research team judged that thematic sufficiency had been reached for the purposes of this study. Given the interpretive and constructivist orientation of the project, we use this judgment not to claim exhaustive coverage of all possible meanings, but to indicate that the dataset was sufficiently rich and coherent to support the three final themes.

To strengthen analytic rigor, coding and theme development were not treated as the product of a single researcher’s unchecked interpretation. Although the first author led the coding process, other members of the research team independently read a subset of transcripts and reviewed emerging codes and thematic groupings. Regular analytic meetings were then held to compare interpretations, question assumptions, and refine theme boundaries. Because this study adopts a reflexive thematic analysis approach, we did not seek statistical inter-coder reliability in the positivist sense. Instead, rigor was enhanced through interpretive dialogue, repeated return to the full dataset, and careful examination of whether the themes remained grounded in participants’ accounts.

Reflexivity was maintained throughout the analytic process. The researchers were aware that their interest in dragon-boat culture and traditional Chinese management philosophy could shape both what they noticed in the data and how they interpreted it. To address this, the first author kept a reflective journal documenting prior assumptions, emotional responses during interviews, and evolving analytic decisions. These reflections were revisited during team discussions to distinguish participants’ meanings from researchers’ theoretical preferences as far as possible. This process helped ensure that the final thematic structure emerged through sustained engagement with the data rather than through premature conceptual imposition.

Additionally, this study strictly adhered to the principles of voluntary participation, anonymity, confidentiality, and the right to withdraw at any time. All participants’ names and team affiliations were anonymized to protect their privacy. The final analytical results are presented in Table 2.

## 3. Results

This critical thematic analysis, based on interview data from twelve dragon-boat helmsters, identifies three core themes: Fade (leading from the rear), Fail-safe (safeguarding the system’s floor), and Fit (attunement to contextual momentum, or shi). Together, these themes underpin what we interpret as a culturally grounded form of leadership, which we term Helmsmanship Leadership.

**Figure 1:**
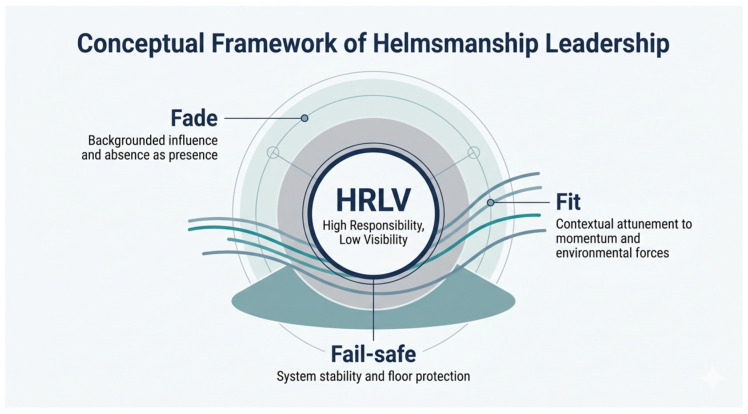
Conceptual Framework of Helmsmanship Leadership.

As summarized in Figure 1, the three themes are analytically distinguishable yet mutually reinforcing. Fade keeps leadership influence backgrounded and minimally intrusive, Fail-safe stabilizes the system by protecting its safety floor and absorbing the burden of error, and Fit enables real-time attunement to the unfolding momentum field. In combination, these dimensions help explain how helmsters sustain collective functioning under conditions of high interdependence, risk, and zero-error tolerance.

### 3.1. Fade

Fade is not simply humility in a generic sense. More precisely, it reflects a Confucian logic of role-embedded restraint in which the leader fulfills a consequential role without converting that role into self-display. Early Confucian texts repeatedly subordinate recognition to adequacy and relational propriety: the junzi worries about lacking capability, not about lacking visibility, and remains dignified without contending. Read through contemporary Confucian role ethics, Fade thus denotes a mode of leadership in which authority is enacted through disciplined self-positioning within the collective rather than through overt self-assertion (Rosemont & Ames, 2016; Ramsey, 2016). Based on our interview data, Fade manifests through five interrelated dimensions. We illustrate two core aspects below.

#### 3.1.1. Invisibility in Perception: Absence as Presence

In dragon-boat racing, the helmster stands alone at the stern, responsible for steering and pacing—a role universally described by participants as “the eyes and the rudder” of the boat. “Only the helmster can see how far we are from the finish line and whether the crew’s rhythm is holding,” one noted. Despite this centrality, the helmster remains perceptually absent.

This invisibility operates on two levels. First, rowers face forward, their backs turned to the helmster; as all participants emphasized, “Except for the drummer, no one sees me.” The helmster thus occupies a paradoxical position: holding critical situational awareness while remaining outside the team’s visual field—functioning as an invisible navigator.

Second, even when interacting with the drummer (who faces the helmster), communication occurs primarily through nonverbal cues. “I give him a hand signal, and he knows exactly where we are,” explained one helmster. These micro-adjustments—through timing, gesture, or rhythm—guide the team without explicit command, embodying Fade’s core logic: influence below the threshold of conscious attention.

During moments of crisis—such as loss of rhythm, low morale, or final sprint—the helmster may issue brief verbal prompts: “It’s falling apart!” or “Push harder in the last 50 m!” Yet these utterances function not as commands but as triggers for collective self-correction. Their purpose is to prevent disintegration, not to assert authority. As such, they exemplify Fade’s “micro-intervention” ethos: minimal input, maximal alignment.

#### 3.1.2. Internalization of Responsibility: Bearing the Emotional Cost of Failure

Fade also entails a distinct moral economy of accountability. Helmsters consistently reported that success is attributed to the team, while failure is internalized as personal fault. “If we win, no one says I steered well. But if we lose, everyone blames me,” stated one participant. This asymmetry reflects a deep-seated ethic: glory flows outward; blame is absorbed inward.

Psychologically, helmsters described feelings of isolation after poor performances, often expressing guilt: “I let the team down,” or “The boat veered—it’s partly my fault.” In contrast, when discussing victories, they spoke only of collective effort: “We shouted together; we pushed as one.” This pattern reveals Fade’s emotional discipline: the leader internalizes negative affect to shield the group, embodying the Confucian ideal of bu zheng (non-contention) and the poetic ethos of “shì liǎo fú yī qù” (“depart quietly once the deed is done”).

Notably, coaches reinforced this dynamic through strategic silence. One coach admitted, “We rarely give feedback to helmsters—even positive reinforcement. Yet they carry immense responsibility.” This absence–presence in institutional discourse subtly normalizes the helmster’s marginality, allowing them to internalize the rear position not as neglect, but as identity. Over time, this structural invisibility becomes a cognitive default, enabling the helmster to operate effectively from the periphery.

Crucially, helmsters grapple with an ethical tension: How to remain invisible yet effective? How to bear cost without seeking recognition? This is the core dilemma of Fade—a leadership stance that achieves impact precisely by refusing center stage.

Together, these practices reveal a leadership form where identity is decoupled from outcome: victory belongs to the collective; failure is privately metabolized. Operating through millisecond-level shifts in timing, angle, or cadence—below the threshold of conscious perception—Fade demonstrates that governance can be both systemically decisive and narratively absent. It challenges the Western equation of leadership with visibility, offering instead a model rooted in restraint, relational ethics, and quiet efficacy.

### 3.2. Fail-Safe

Fail-safe is a conceptually innovative leadership principle that prioritizes establishing a system’s safety floor over pursuing performance breakthroughs. It precisely captures the helmster’s core value: not to achieve heroic “ceiling-breaking” feats—those arise from the crew’s collective effort—but to absolutely prevent catastrophic “floor breaches” such as veering off course or capsizing. Success for the helmster is defined by safety, stability, and error avoidance, providing the invisible foundation upon which team excellence rests. This embodies a strategic wisdom rooted in prevention-first and steady progress through resilience.

Our analysis of interview data reveals two interrelated sub-dimensions through which Fail-safe operates.

#### 3.2.1. Role Identity: Guardian, Not Breakthrough Agent

All participants described the helmster as a system guardian, not a performance driver. As one stated:

“My ceiling is very clear—I can’t make the boat significantly faster in a major race through my own skill. All I do is protect the floor: I keep it from going off track.”

Another added:

“Helmster technique isn’t visible; it won’t shave seconds off the time, but its core function is to prevent drift.”

The helmster’s mandate is to “ensure the team crosses the finish line safely”—not to issue commands or design strategy. One explained the physical logic:

“I must first stabilize my own body. As long as I stay on the stern, the boat rarely flips. But if I fall, it almost certainly capsizes.”

During training, helmsters monitor crew coordination and offer subtle corrections:

“If I see a rower in one section struggling, I’ll quietly alert them on the boat.”

These practices reflect a preventive, embedded role: the helmster enables high performance by eliminating systemic risks, not by leading overtly. Crucially, helmsters operate under constant fear of error—and bear full accountability when mistakes occur. This reinforces their identity as risk absorbers, whose legitimacy stems from flawless execution, not charismatic intervention.

#### 3.2.2. Emotional Accountability: Bearing the Cost of Floor Collapse

When the team fails to medal, “the helmster suffers most—not the rowers,” noted one participant. Another emphasized:

“We’re expected to deliver a mistake-free race. If an error happens, it’s 100% my fault.”

This emotional burden aligns with Fail-safe’s core ethic: the guardian internalizes the cost of system failure.

One helmster reflected on the essential quality for the role:

“Courage. Not the courage to excel, but the courage to stand up—and to bear total responsibility.”

This is not the Western ideal of “courage to inspire,” but a stoic readiness to be the last line of defense.

Collectively, these findings position the helmster as the boundary sentinel of the team: their existence ensures the system remains within safe operational limits. Authority here is not derived from delegation or charisma, but from precise attunement to the boat’s rhythm and balance.

We therefore interpret Fail-safe through wu-wei with greater precision than “non-action.” The helmster clearly acts, but acts in ways that minimize friction, avoid theatrical overcorrection, and preserve the integrity of the whole system. This understanding is closer to the classical Chinese ideal of effortless and non-coercive efficacy than to passivity in any literal sense (Slingerland, 2007).

It also resonates with the Daodejing’s ideal of accomplishing work without claiming possession of it or dwelling on achievement, and with the Analects’ image of governing effectively through properly inhabiting one’s role rather than through conspicuous intervention. In organizational terms, Fail-safe is a form of non-coercive efficacy: leadership works by stabilizing the conditions under which the collective can function reliably.

### 3.3. Fit

Fit denotes a shi-based leadership logic in which the helmster does not merely adapt to situational conditions, such as wind, waves, water currents, crew fatigue, or race phase, but continuously attunes to an emergent momentum field composed of these shifting forces. In this sense, shi refers not simply to “context” in a static sense, but to the propensity, configuration, and directional potential immanent in a situation and available for strategic leverage (Jullien, 1999). It embodies the Chinese strategic ethos of “going with the flow” (shun shi er wei) and “timely adaptation” (yu shi ju jin) (Mott & Kim, 2006).

Authority under Fit is provisional and context-contingent: it emerges only when the helmster’s actions achieve resonance with the changing configuration of tian shi (timing), di li (terrain), and ren he (team harmony). In practice, this means not simply responding flexibly to a given situation, but sensing subtle changes as they unfold and recalibrating action from within the situation itself. External forces are therefore treated not merely as obstacles to be managed, but as potential sources of propulsion that can be tactically incorporated into the boat’s movement.

#### 3.3.1. Sensing and Harnessing Situational Momentum

Our analysis reveals two analytical aspects through which Fit operates:

Harnessing Environmental Momentum: Turning Wind, Waves, and Currents into Advantage.

Participants consistently described the helmster as both the boat’s “rudder” and its “eyes,” requiring deep intuitive knowledge of hydrodynamics: “You must understand the water and read the wind.” One helmster illustrated this with precision:

“If a neighboring boat is faster, the wave it creates moves forward. An experienced helmster can ‘borrow’ that wave—using it to swing the dragon head just right—and gain speed.”

This account captures the core logic of Fit. Rather than resisting the wake of another boat, the helmster senses its direction and force, then incorporates that external energy into the boat’s own trajectory. The goal is not to dominate the environment through control, but to move with its rhythms and convert even competitive pressure into a resource.

Effective ‘Fit’ leadership begins with an embodied, holistic perception. Unlike rule-based steering, it relies on an intuitive understanding of wind and water, forged through long-term practice. This distinction becomes most evident under adverse weather conditions:

“Rainy conditions are a huge test with chaotic winds and waves. We have to really feel the boat’s center of gravity and the water current. In one race, we stuck to our game plan and secured the win.”

This narrative underscores that true mastery lies not in resisting the elements, but in harmonizing with them—transforming environmental volatility into a strategic asset. Ultimately, the helmster’s ability to ‘fit’ the context allows the team to navigate uncertainty with resilience, turning potential chaos into a clear path toward victory.

The core distinction between novice and veteran helmsters lies in their ability to anticipate momentum rather than merely react to it:

“Novices fixate on the lane, only correcting the helm when the boat drifts. Veterans, however, read the environment to adjust proactively. It’s like driving: novices slam on the brakes at the sight of an obstacle, while veterans anticipate and glide through with composure.”

Ultimately, this distinction transcends mere technique—it is a fundamental shift in mindset. The veteran helmster does not fight the water but flows with it, turning potential hazards into navigable paths through foresight. This is the essence of mastery: not reacting to the present, but shaping the future.

#### 3.3.2. Processual Authority Through Contextual Attunement

Importantly, this is more than generic contextual flexibility. Participants did not describe the race environment as a fixed backdrop to which the helmster simply “adjusts.” Instead, they portrayed it as a moving field of forces—wind, current, wake interference, crew rhythm, and race phase—that must be read in real time. Fit therefore operates less through choosing from a repertoire of pre-formed responses than through ongoing embodied micro-calibration, rather than the style–situation matching more commonly associated with situational leadership frameworks (Fiedler, 1971; Graeff, 1997).

Such accounts point to a distinct epistemology of action: efficacy arises not from imposing order on the situation, but from perceiving, absorbing, and redirecting situational momentum (shi). The helmster’s authority derives not from title or charisma, but from demonstrated competence in attuning to this emergent field and acting in step with it, rather than from the leader-centered performative influence emphasized in transformational models (Bass, 1985).

Under Fit, the leader functions less as a commander than as a conduit between team and environment. Their primary task is not to alter conditions wholesale, but to detect subtle shifts and reconfigure action accordingly—turning headwinds into lift, rival wakes into thrust, and instability into navigable movement. In this sense, Fit reflects a more ecologically embedded and relationally emergent form of leadership in fluid systems (Uhl-Bien et al., 2007; Wielkiewicz & Stelzner, 2005).

Crucially, authority here is ephemeral and earned anew in each moment. It exists only to the extent that the helmster’s intervention remains faithful to the demands of the unfolding situation. Fit thus reveals a processual legitimacy grounded not in stable personal traits or fixed positional power, but in real-time contextual attunement.

In sum, Fit highlights a form of leadership in which agency lies not in forcing outcomes through unilateral control, but in sensing momentum and moving with it. In fluid, interdependent systems, effective leadership may depend less on making things happen than on enabling the right movement to emerge through timely, situated, and ecologically intelligent action.

## 4. Discussion

Drawing from the extreme yet archetypal practice field of Chinese dragon-boat helmsters, this study develops Helmsmanship Leadership, characterized by High Responsibility and Low Visibility (HRLV).

In this sense, Helmsmanship Leadership is intended not merely as an interesting local description, but as a theoretically generative concept emerging from the dragon boat context. This responds to longstanding calls in management and organization scholarship for indigenous theorizing that begins from local categories, practices, and philosophical assumptions, rather than treating non-Western settings as sites for the secondary testing of Western frameworks (Tsui, 2004; Li et al., 2012). It also extends efforts to connect traditional Chinese philosophies with contemporary leadership theorizing in a more empirically grounded manner (Ma & Tsui, 2015).

This model helps illuminate a relatively underexplored aspect of leadership research: mainstream theories have long been dominated by the “high-visibility, high-responsibility” heroic narrative (Meindl et al., 1985), while giving less attention to governance actors who bear substantial accountability yet deliberately recede from view.

High Responsibility and Low Visibility leadership style, such as transformational leadership (Bass & Riggio, 2006) and charismatic leadership (Conger & Kanungo, 1998) have typically equated effective leadership with visible vision articulation, personal magnetism, and performance breakthroughs.

In contrast, helmster practice reveals an alternative logic: true control sometimes lies precisely in making control imperceptible; peak efficacy often emerges from deepest invisibility. Although adjacent constructs such as quiet and invisible leadership, silent coordination, HRO, and sensemaking each illuminate parts of this phenomenon foregrounding restrained influence, implicit alignment, reliability, and order under uncertainty (Badaracco, 2002; Alvesson & Sveningsson, 2003; Rico et al., 2008; Weick, 1993; Sutcliffe, 2023), they do not fully theorize a leadership position in which public credit is systematically minimized while accountability becomes institutionally concentrated in a focal role within a relational high-reliability system.

The HRLV framework should therefore not be reduced to a cultural curiosity; rather, as summarized in Figure 1, within this case it appears as a coherent, interlocking, and adaptive configuration constituted by three synergistic dimensions: Fade enables silent coordination, Fail-safe anchors the system’s floor, and Fit supports dynamic attunement to context.

It is this systemic coupling that allows helmsters to sustain collective functioning under conditions of zero-error tolerance, full interdependence, and physical inversion, where every rower faces forward, literally turning their backs on the leader.

Importantly, HRLV should not be understood as a purely individual attribute of the helmster. Rather, it emerges through a relational high-reliability arrangement in which the boat’s spatial architecture, forward-facing crew formation, drummer–helmster signaling, collective attribution patterns, and zero-error expectations jointly produce low visibility and high responsibility. In this sense, HRLV is relationally produced, even though it becomes institutionally concentrated in the helmster role.

### 4.1. Fade: Leading Through Absence

Positioned at the stern, the helmster is physically obscured behind the crew, rarely speaks, and exercises authority not through explicit commands but via sub-threshold interventions: millimeter-level rudder adjustments, synchronized breathing, or micro-timing cues that silently steer the boat’s direction and rhythm.

Notably, due to their distinct skill set—non-rowing, non-participatory in technical drills—the helmster often experiences structural solitude during training: while others gather to discuss technique, the helmster sits apart, socially peripheral yet functionally central.

This individual, though seemingly marginal, serves as the team’s “rudder” and “eyes,” bearing ultimate responsibility for safety and synchronization. The coexistence of high accountability and low social embeddedness epitomizes the HRLV condition. Crucially, leadership efficacy here is not positively correlated with perceptibility—in fact, it may be inversely related: the less the intervention is noticed, the smoother the collective flow.

This finding invites reconsideration of the common assumption that “visibility equals effectiveness” (Bass & Riggio, 2006), which often associates leadership with vocal visioneering and charismatic presence. It also diverges from servant leadership’s emphasis on perceivable care and emotional availability (Greenleaf, 1977). Instead, helmster practice offers an embodied interpretation of Daoist wu-wei (non-action): governance can operate by allowing intervention to dissolve into collective action itself.

As the Daodejing (Chapter 2) states:

“When the work is done, the people say, ‘We did it ourselves.’”

(Gong cheng fu ju; fu wei fu ju, shi yi bu qu.)

In this light, Helmsmanship Leadership suggests a way of understanding influence not as imposition, but as invisible enablement; not primarily as being seen, but as helping make collective success possible without being seen.

### 4.2. Fail-Safe: Preventive Authority and Systemic Floor Protection

The helmster’s core mission is not to win but to ensure the boat does not capsize. Their success criterion is “nothing happening”—a form of preventive authority (Sutcliffe, 2020) marked by the absence of disaster. This contrasts sharply with Burns’ (1978) transformational leadership, which aims to inspire transcendence beyond self, and Weick’s (1993) focus on post-crisis sensemaking. The helmster’s primary role is maintaining order and stability, repositioning the leader from a “growth engine” to a systemic safety net.

This “floor protection” logic embodies a profound anti-miracle ethic: in zero-tolerance environments, avoiding failure is the highest achievement. It underscores the strategic value of building disaster-resilient floors in increasingly fragile complex systems—perhaps more critical than chasing performance ceilings.

### 4.3. Fit: Situational Attunement and Shaped by Momentum

Helmsters do not adhere rigidly to pre-set courses but instead respond dynamically to currents, winds, and wakes from other boats, leveraging external forces to enhance performance. This capacity for situational attunement is rooted in the strategic philosophy of Sun Tzu’s Art of War: “Skilled warriors seek advantage through situational momentum” (Mott & Kim, 2006). Authority under Fit fluctuates with the situation—emerging in adversity, receding in ease. This is different from what is typically emphasized in contingency theory’s (Fiedler, 1971) focus on static matching and differs from complexity leadership theory’s (Uhl-Bien et al., 2007) view of leaders as interventionists. Helmsters see themselves as part of the environment, pursuing co-evolution with context—an embodiment of shi-based agency. Decisions are based on intuitive grasp of the overall “momentum field,” rather than isolated variables, thereby offering an empirically grounded illustration of a more holistic mode of organizational action.

This work encourages scholars to shift more attention from the spotlight to the shadows, where important forms of leadership may also reside. Dragon-boat helmsters are not simply isolated cultural phenomena, but a case that helps reveal some limits in dominant leadership assumptions. They suggest that leadership may often be contextual, culturally specific, and decentralized. While mainstream theories have often centered the “high visibility, high accountability” heroic narrative (Meindl et al., 1985), helmsmanship leadership indicates that consequential governance can also occur outside the limelight, and that authority may sometimes arise from the deliberate relinquishment of personal glory.

This practice-based insight—rooted in the physical and cultural soil of dragon-boat racing and developed into a theoretically suggestive framework—offers a useful lens for understanding management practices in Chinese organizations and contributes a valuable perspective from an Eastern context to efforts aimed at building more culturally inclusive and explanatory leadership theories (House et al., 2004; Dorfman et al., 2012). Ultimately, it calls us to listen to the voices of practice from diverse locales (Escobar, 2001), while recognizing that leadership concepts remain shaped by the empirical grounds from which they emerge.

## 5. Conclusions

This study examined leadership practice in dragon-boat racing and developed an interpretive framework centered on Helmsmanship Leadership. Based on interviews with helmsters, we identified three interrelated themes—Fade, Fail-safe, and Fit—that together describe a form of leadership marked by low visibility, concentrated responsibility, preventive vigilance, and situational attunement.

The contribution of the study is therefore primarily conceptual and exploratory. Rather than proposing a universally applicable new paradigm, the paper shows how an extreme case can help make visible aspects of leadership that are less emphasized in mainstream theories, especially when effective action is enacted through backgrounded influence, the protection of system stability, and close alignment with contextual momentum. In this sense, the study contributes a culturally grounded perspective that may complement broader leadership scholarship and encourage closer attention to forms of leadership that are understated, relationally embedded, and context-dependent.

At the same time, the claims of this study should remain proportionate to its evidence base. The findings derive from a single cultural setting, a small purposive sample, and qualitative interview data. Their value lies less in universal generalization than in opening a theoretically suggestive line of inquiry. Future research is needed to examine whether, how, and to what extent similar dynamics emerge in other organizational and cultural contexts.

## 6. Strengths

This study offers 3 significant contributions to the field of leadership research:

(1) By drawing on the extreme and archetypal practice of Chinese dragon-boat helmsters, this research successfully conceptualizes “Helmsmanship Leadership”—a coherent and holistic leadership model defined by its high-responsibility, low-visibility (HRLV) core. Comprising three interlocking dimensions—Fade, Fail-safe, and Fit—this paradigm offers a new alternative to the dominant high-responsibility, high-visibility leadership style, which emphasizes visibility, heroism, and the relentless pursuit of performance ceilings.

(2) Bridging Eastern Philosophy and Western Theory. The study translates classical Chinese philosophical resources—especially Confucian role ethics, Daoist wu-wei, and shi-based strategic thinking—into an empirically grounded leadership framework. Rather than treating Chinese philosophy as a cultural backdrop, the paper uses these traditions as theoretical micro-foundations for explaining relational authority, non-coercive coordination, and situational attunement.

(3) Providing a culturally grounded perspective for leadership scholarship. By centering a non-Western, relationally oriented context, this research shows how authority may be enacted through preventive guardianship, contextual attunement, and low-visibility responsibility. In doing so, it adds empirical and conceptual nuance to ongoing debates about the cultural embeddedness of leadership.

## 7. Limitations

Despite its contributions, this study has 2 limitations that warrant acknowledgment:

(1) Contextual Specificity of the Empirical Setting. The findings are derived exclusively from the highly specialized and ritualized context of competitive Chinese dragon-boat racing. While this setting serves as an ideal “extreme case” for theory generation, the direct applicability of the Helmsmanship Leadership model to other organizational contexts (e.g., corporate, political, or virtual teams) remains an open question and requires empirical validation.

(2) Reliance on a Single-Case, Qualitative Design. As a qualitative study based on in-depth interviews with a purposive sample of helmsters, the research prioritizes theoretical depth over statistical generalizability. The rich, contextual data provide strong analytical insights, but the findings may not be representative of all helmsters or leadership practices in other high-stakes, interdependent teams.

## 8. Future Research Directions

Building on this foundational work, we propose 4 promising avenues for future research:

(1) Cross-Contextual Validation and Application. Future studies should test the Helmsmanship Leadership framework in diverse organizational settings beyond sports, such as crisis management teams, surgical units, or software development squads—anywhere where HRLV dynamics, systemic safety, and silent coordination are paramount. This would assess the model’s transferability and practical utility.

(2) Quantitative and Mixed-Methods Approaches. To complement this qualitative foundation, researchers could develop scales to measure the three dimensions (Fade, Fail-safe, Fit) and conduct large-sample surveys or experiments. This would allow for hypothesis testing, examination of the model’s nomological network, and assessment of its predictive power for team performance and resilience.

(3) Cross-Cultural Comparative Studies. A critical next step is to conduct comparative research in non-Chinese cultural contexts. Investigating similar “helmster-like” roles in other cultures (e.g., coxswains in Western rowing, conductors in orchestras, or project managers in Nordic consensus-driven firms) would help disentangle what aspects of Helmsmanship Leadership are culturally specific versus universally applicable, thereby refining the model’s cross-cultural validity.

(4) Exploring the Role of Gender and Diversity. Future research should explicitly examine how gender, age, and other diversity factors interact with the Helmsmanship Leadership model. Understanding how these dimensions are enacted and perceived by leaders from different backgrounds will provide a more nuanced and inclusive understanding of this leadership paradigm.

## Figures and Tables

**Table 1:** The information of the participants.

Code	Age	Gender	Major	Participation Duration (Years)
Male 1	19	Male	Public Finance	2
Male 2	21	Male	Chemistry	3
Male 3	23	Male	Marketing Management	2
Male 4	23	Male	Economics	2
Male 5	24	Male	Architectural Engineering	1
Female 1	21	Female	Mathematics and Applied Mathematics	4
Female 2	21	Female	Physics Science	4
Female 3	21	Female	Communication Studies	4
Female 4	21	Female	Management Science	4
Female 5	21	Female	English Language and Literature	4
Female 6	20	Female	English Language and Literature	3
Female 7	20	Female	Archaeological Science	3

**Table 2:** Illustrative Excerpts, Coding Progression, and Final Themes.

Raw Excerpt	Initial Code	Focused Code	Theme
If we win, no one praises me; if we lose, all blame me.	Asymmetric credit-blame	Internalize failure	Fade
Victory is the team’s; I just do my part	Collective attribution	No personal claim	
Only the drummer sees me; I use hand signals.	Non-visible presence	Implicit influence	
I remind, not command; let them self-correct.	Minimal intervention	Sub-threshold guidance	
I cannot speed up; only keep safe & straight.	Role boundary	System guardian	Fail-safe
Helmster’s mistake is full responsibility.	Zero error tolerance	Absolute accountability	
If I stand firm, boat won’t flip.	Personal system stability	Bottom-line protection	
My success is nothing bad happens.	No disaster	Prevention orientation	
Must read water and wind; adjust to conditions.	Environmental sensing	Sense momentum (shi)	Fit
Experienced helmsters borrow waves for speed.	Use external force	Convert momentum	
Novices brake in panic; veterans anticipate with ease.	Dynamic matching	Rhythm alignment	
Good turn preserves speed by following water flow.	Flow-based turning	Momentum-conserving action	
Compensate for crew imbalance quietly.	Internal balance adjustment	Integrate internal & external shi	

## Data Availability

The data presented in this study are available on request from the corresponding author due to ethical and privacy concerns. Several participants expressed strong reservations about public disclosure of their interview transcripts, particularly regarding sensitive personal reflections—such as feelings of isolation, emotional vulnerability, and relational tensions within the team—and explicitly requested that the raw data not be made publicly accessible.

## References

[B1-behavsci-16-00710] Alvesson M., Sveningsson S. (2003). The great disappearing act: Difficulties in doing “leadership”. The Leadership Quarterly.

[B2-behavsci-16-00710] Badaracco J. (2002). Leading quietly: An unorthodox guide to doing the right thing.

[B3-behavsci-16-00710] Bass B. M. (1985). Leadership and performance beyond expectations.

[B4-behavsci-16-00710] Bass B. M., Riggio R. E. (2006). Transformational leadership.

[B5-behavsci-16-00710] Braun V., Clarke V. (2019). Reflecting on reflexive TA. Reflexive thematic analysis for health researchers.

[B6-behavsci-16-00710] Burns J. M. (1978). Leadership.

[B7-behavsci-16-00710] Collinge C., Gibney J. (2010). Connecting place, policy and leadership. Policy Studies.

[B8-behavsci-16-00710] Collinge C., Gibney J., Mabey C. (2010). Leadership and place.

[B9-behavsci-16-00710] Conger J. A., Kanungo R. N. (1998). Charismatic leadership in organizations.

[B10-behavsci-16-00710] Crotty M. (1998). The foundations of social research: Meaning and perspective in the research process.

[B11-behavsci-16-00710] Day D. V. (2014). Advances in leadership and leadership development: A review of 25 years of research and theory. The Leadership Quarterly.

[B12-behavsci-16-00710] Dorfman P., Javidan M., Hanges P., Dastmalchian A., House R. (2012). Globe: A twenty year journey into the intriguing world of culture and leadership. Journal of World Business.

[B13-behavsci-16-00710] Eisenhardt K. M., Graebner M. E. (2007). Theory building from cases: Opportunities and challenges. Academy of Management Journal.

[B14-behavsci-16-00710] Escobar A. (2001). Culture sits in places: Reflections on globalism and subaltern strategies of localization. Political Geography.

[B15-behavsci-16-00710] Farh J. L., Cheng B. S. (2000). A cultural analysis of paternalistic leadership in Chinese organizations.

[B16-behavsci-16-00710] Fiedler F. E. (1971). Validation and extension of the contingency model of leadership effectiveness: A review of empirical findings. Psychological Bulletin.

[B17-behavsci-16-00710] Flyvbjerg B. (2004). Five misunderstandings about case-study research. Sosiologisk Tidsskrift.

[B18-behavsci-16-00710] Graeff C. L. (1997). Evolution of situational leadership theory: A critical review. The Leadership Quarterly.

[B19-behavsci-16-00710] Greenleaf R. K. (1977). Servant leadership: A journey into the nature of legitimate power and greatness.

[B20-behavsci-16-00710] House R. J. (1976). A 1976 theory of charismatic leadership.

[B21-behavsci-16-00710] House R. J., Hanges P. J., Javidan M., Dorfman P. W., Gupta V. (2004). Culture, leadership, and organizations: The GLOBE study of 62 Societies.

[B22-behavsci-16-00710] Jullien F. (1999). The propensity of things: Toward a history of efficacy in China.

[B23-behavsci-16-00710] Kirkman B. L., Lowe K. B., Gibson C. B. (2006). A quarter century of culture’s consequences: A review of empirical research incorporating hofstede’s cultural values framework. Journal of International Business Studies.

[B24-behavsci-16-00710] Kvale S., Brinkman S. (2009). InterViews: Learning the craft of qualitative research interviewing.

[B25-behavsci-16-00710] Li P. P., Leung K., Chen C. C., Luo J.-D. (2012). Indigenous research on Chinese management: What and how. Management and Organization Review.

[B26-behavsci-16-00710] Ma L., Tsui A. S. (2015). Traditional Chinese philosophies and contemporary leadership. The Leadership Quarterly.

[B27-behavsci-16-00710] Mabey C., Freeman T. (2010). Reflections on leadership and place. Policy Studies.

[B28-behavsci-16-00710] Maitlis S., Christianson M. (2014). Sensemaking in organizations: Taking stock and moving forward. Academy of Management Annals.

[B29-behavsci-16-00710] Meindl J. R., Ehrlich S. B., Dukerich J. M. (1985). The romance of leadership. Administrative Science Quarterl.

[B30-behavsci-16-00710] Mott W. H., Kim J. C. (2006). Chinese strategy: Shih-strategy. The philosophy of Chinese military culture: Shih vs. Li.

[B31-behavsci-16-00710] Nor N. A., Wuen C. H., Ismail A. (2017). Leadership style desired by youth in Asia. The Journal of Management Development.

[B32-behavsci-16-00710] Palinkas L. A., Horwitz S. M., Green C. A., Wisdom J. P., Duan N., Hoagwood K. (2015). Purposeful sampling for qualitative data collection and analysis in mixed method implementation research. Administration and Policy in Mental Health and Mental Health Services Research.

[B33-behavsci-16-00710] Pfeffer J. (1977). The ambiguity of leadership. Academy of Management Review.

[B34-behavsci-16-00710] Ramsey J. (2016). Confucian role ethics: A critical survey. Philosophy Compass.

[B35-behavsci-16-00710] Rico R., Sánchez-Manzanares M., Gil F., Gibson C. (2008). Team implicit coordination processes: A team knowledge–Based approach. Academy of Management Review.

[B36-behavsci-16-00710] Rosemont H., Ames R. T. (2016). Confucian role ethics: A moral vision for the 21st century?.

[B37-behavsci-16-00710] Slingerland E. (2007). Effortless action: Wu-wei as conceptual metaphor and spiritual ideal in early China.

[B38-behavsci-16-00710] Sutcliffe K. M. (2020). High reliability organizing: Managing collective mindfulness. Oxford research encyclopedia of business and management.

[B39-behavsci-16-00710] Sutcliffe K. M. (2023). Building cultures of high reliability: Lessons from the high reliability organization paradigm. Anesthesiology Clinics.

[B40-behavsci-16-00710] Tsui A. S. (2004). Contributing to global management knowledge: A case for high quality indigenous research. Asia Pacific Journal of Management.

[B41-behavsci-16-00710] Uhl-Bien M., Marion R., McKelvey B. (2007). Complexity leadership theory: Shifting leadership from the industrial age to the knowledge era. The Leadership Quarterly.

[B42-behavsci-16-00710] Weick K. E. (1993). The collapse of sensemaking in organizations: The Mann Gulch disaster. Administrative Science Quarterly.

[B43-behavsci-16-00710] Weick K. E., Sutcliffe K. M., Obstfeld D. (1999). Organizing for high reliability: Processes of collective mindfulness, research in organizational behaviour. Research in Organizational Behavior.

[B44-behavsci-16-00710] Wielkiewicz R. M., Stelzner S. P. (2005). An ecological perspective on leadership theory, research, and practice. Review of General Psychology.

[B45-behavsci-16-00710] Wright E., Walker A., Bryant D., Lee M., Hassan K. S., Choi S. (2021). A cross-cultural study of student leadership in round square schools.

